# Developmental Changes in Pain and Spinal Immune Gene Expression after Radicular Trauma in the Rat

**DOI:** 10.3389/fneur.2016.00223

**Published:** 2016-12-15

**Authors:** Gordon A. Barr, Shaoning Wang, Christine L. Weisshaar, Beth A. Winkelstein

**Affiliations:** ^1^Division of Basic Science Research, Department of Anesthesiology and Critical Care Medicine, The Children’s Hospital of Philadelphia, Perelman School of Medicine at the University of Pennsylvania, Philadelphia, PA, USA; ^2^Spine Pain Research Laboratory, Department of Bioengineering, School of Engineering and Applied Science, University of Pennsylvania, Philadelphia, PA, USA

**Keywords:** neuropathic pain, compression, ontogeny, immune, cytokines, chemokines, hyperalgesia, allodynia

## Abstract

Neuropathic pain is chronic pain that develops after nerve injury and is less frequent in infants and children than in adults. Likewise, in animal models of neuropathic pain, allodynia and hyperalgesia are non-existent or attenuated in the infant, with a “switch” during development by which acute nerve injury transitions to chronic pain. Concomitant with the delay in neuropathic pain, there is a parallel delay in the ability of nerve injury to activate the immune system. Models of neuropathic pain in the infant have used various ligation methods and find that neuropathic pain does not occur under after postnatal days 21–28 (PN21–PN28), linked to activation of immune processes and developmental regulation of anti-inflammatory cytokines. We applied a model of neuropathic pain in the adult using a transient compression of the cervical nerve or nerve root in infant rats (injured at 10, 14, 21, or 28 days of age) to define transition periods during which injury results in no change in thermal and mechanical pain sensitivity or in short-term changes in pain. There was little to no hyperalgesia when the injury was imposed at PN10, but significant thermal hyperalgesia and mechanical allodynia 1 day after compression injury when performed at PN14, 21, or 28. Thermal withdrawal latencies returned to near baseline by 7 days postsurgery when the injuries were at PN14, and lasted up to 14 days when the injury was imposed at PN28. There was mechanical allodynia following injury at 1 day postinjury and at 14 days after injury at PN14. Measurements of mRNA from spinal cord at 1, 7, and 14 days postinjury at PN14, 21, and 28 showed that both the magnitude and duration of elevated immune markers and chemokines/cytokines were greater in the older animals, corresponding to the development of hyperalgesia. Thus, we confirm the late onset of neuropathic pain but found no evidence of emergent hyperalgesia if the injury was before PN21. This may be due to the use of a transient, and not sustained, compression ligation model.

## Introduction

As with all sensory systems, nociceptive circuits are plastic in infancy. Neonates respond to acute painful stimulation and show hyperalgesia after inflammatory injury at or before birth (1–6). Nonetheless nociceptive circuits and resultant pain processes develop and change well into postnatal life (1, 7–13). Neuropathic pain following peripheral nerve damage is one example of a process that is immature in the infant. Phantom limb pain exists in children, but the incidence is 10-fold less than for adults. Complex regional pain syndrome is rare until adolescence. Brachial plexus avulsion often produces severe and debilitating pain in adults but not when it occurs obstetrically (14–16). Similarly in the rodent infant, regardless of the injury model or the age at which the injury occurs, changes in pain thresholds appear only when tested at 21–33 days of age [(17–27); see Table 1]. In some studies, there was a delay between injury and decreased thresholds or an earlier resolution of the pain (19, 21, 27). Thus the increased propensity to develop neuropathic pain does not appear until early adolescence in both the rat and human [reviewed in Ref. (14–16, 18)].

**Table 1:** **Summary of published peripheral nerve injury studies on pain during development**.

Model	Age	Short-term pain	Long-term pain	Mechanism	Reference
Caudal trunk transection	PN0 PN10 Adult	No effect for PN0 or PN10 Immediate for adult	Appeared at 4–6 weeks postinjury in PN0 and PN10		(21)

C-fiber stimulation	PN10 Adult	Allodynia 3–48 h in adult but not PN10	Not tested	Spinal microglia activation only in adult	(23)

L5, L6 ligation	PN7 PN14 PN21	Appeared when tested at PN21 regardless of age of lesion	Resolved at 6–8 weeks for PN7 and PN14 but not PN21		(19)

Partial sciatic (Seltzer) Spinal nerve (Chung)	PN14 PN28 Adult	PSL – no effect at PN14 SNL – all ages allodynia 1 week postinjury (PN21, PN35)	PN14 resolved 4–6 weeks PN28 resolved 7 weeks Adult resolved 8 weeks		(27)

Spared nerve injury (SNI) Chr. constriction (PN10 only)	PN3 PN10 PN21 PN28 PN33	SNI-PN3 no effect PN10 and 21 show non-specific transient allodynia. 7 days postop PN33 allodynic	Only PN33 shows long-term effect No reappearance		(20)

SNI i.t. NMDA, LPS, or activated microglia	PN10 PN21	Not tested for SNI LPS produces small but significant allodynia in PN10 and PN21 Activated microglia had no effect	Not tested	Spinal microglial markers less elevated at PN10 SNI NMDA and LPS elevated microglial markers at both ages	(22)

SNI i.t. LPS i.t. ATP activated microglia	PN3 PN10 PN21 Adult	Not reported	Not tested	Adult spinal microglia (3 days) and astrocyte (5 days) activation Infant microglia weak but early (1 day) robust astrocyte activation	(24)

SNI	PN10 Adult	No immediate allodynia at PN10	Not tested	Genes related to immune function activated only in adult DRG Macrophages cluster around A-fiber cell bodies only in adult	(25)

SNI	PN10 Adult	No allodynia before PN21	Appeared only at PN33 after PN10 injury	T-cells infiltrate the spinal cord in adults not infants Identify different genes expressed in adults vs. infants related to immune response	(17)

SNI	PN10 PN35 Adults	No immediate allodynia at PN10	Cold, mechanical, and weight bearing changes only after PN30. No thermal changes	Upreg. of selective immune markers Anti-inflammatory IL-4 and IL-10 cytokines are protective in infants	(48)

SNI Minocycline or ketamine treatment	PN10	No immediate allodynia at PN10	Mechanical allodynia only after PN31. No thermal changes	Allodynia accompanied by macrophage, microglial, and astrocyte activation NMDA dependent	(72)

There are multiple mechanisms that have been proposed to be responsible for neuropathic pain [see Ref. (18, 28–36) for examples and reviews]. Injury activates both the innate (37–41) and adaptive immune (17, 41–44) systems and dampening the immune response reduces pain (45, 46). Thus, the immune system is involved in the initiation and maintenance of neuropathic pain following peripheral or central nervous system damage (29, 47). These immune processes may maintain neuropathic pain, which are then amenable to immune suppressant drugs. The mechanisms that induce and maintain neuropathic pain that are immature in the infant, or that may be protective, are largely unknown, although recently two inhibitory cytokines, IL-4 and IL-10 were overexpressed in the infant and protective, inhibiting neuropathic pain [(48); reviewed in Ref. (18)]. Concurrent with the lack of neuropathic pain is the limited ability of nerve injury to activate immune markers in the spinal cord or DRG (19–22, 24, 25, 27). We do not fully know why the immune response to nerve damage is immature. Immune activation in neonates can be induced by other insults (e.g., ischemic brain injury; i.t. NMDA, *Escherichia coli* or LPS injection; and intraplantar carrageenan injection). It is not therefore, the inability of the immune system to respond but rather the inability of neural injury to activate the immune system.

**Figure 1:**
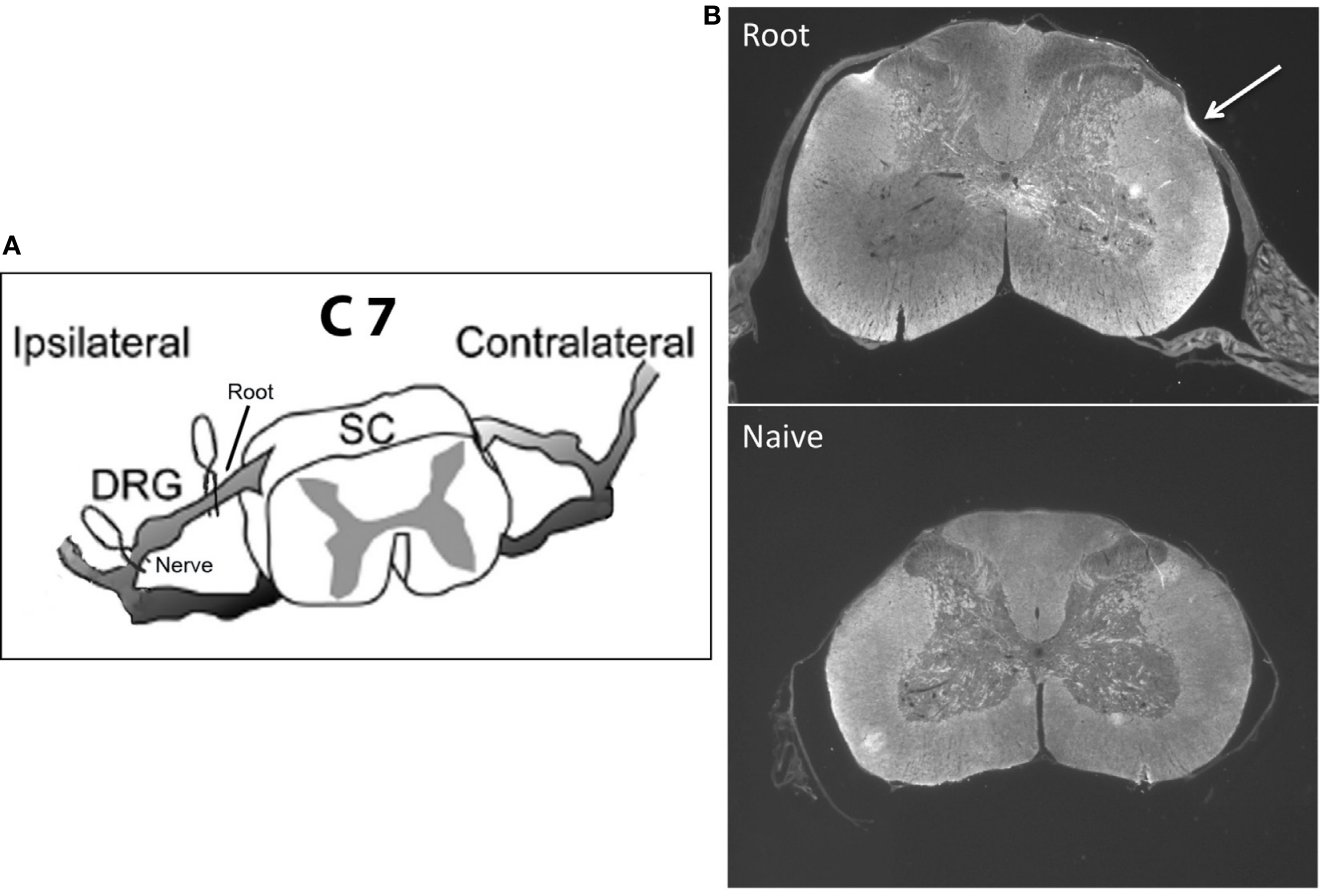
**(A)** shows a schematic of the location of the microclips distal (nerve compression) and proximal (root compression) to the DRG. The nerve root compression was applied to the dorsal root as it exited the DRG. The nerve compression was of the mixed nerve just prior to its entering the DRG. **(B)** is a sample photomicrograph of myelin basic protein staining 14 days following root compression in a 28-day old animal (top) and a same age control (bottom). The arrow denotes the site of compression. We use these figures only to show the nature of the compression injury and not to imply any quantitative changes.

The specific goal here is to understand the mechanisms that protect the infant from developing chronic neuropathic pain and how those mechanisms change as the infant matures. Studies of neuropathic pain in the infant have focused on peripheral nerve injury using a variety of models, almost all of which use some form of permanent/sustained nerve ligation. We have adapted rat models of transient cervical nerve or root compression and applied them to infants (Figure 1A). This avoids potential interactions of ligation with nerve growth during early development and has not been tested in infants. In adult rodents, cervical root compression, a transient one-time event, induces long-term thermal and mechanical hyperalgesia (49, 50). In humans, the comparable injury is brachial plexus avulsion. To test the maturation of the behavioral and immune responses to this more transient injury, we induced the compression injury at 10–28 days of age and assessed pain responses 1, 7, and 14 days later to bracket ages at which there are no effects on pain thresholds and when neuropathic pain first appears (19–27). In addition, we measured mRNA for immune related markers and cytokines at those time points. We found that the injury produced no neuropathic pain in infants, short-term allodynia, and hyperalgesia that resolves quickly in weanlings and a longer lasting change in thermal pain thresholds in juveniles. Furthermore, the number, intensity, and duration of immune mRNA responses induced by injury increased with age and were greater following root injury than nerve injury. Our working hypotheses were that (1) nerve root compression activates the immune system, which is necessary but not sufficient to produce long-term pain; and (2) this activation follows the developmental course of neuropathic pain.

## Materials and Methods

### Subjects

Subjects were male and female Long–Evans rats from Harlan Labs (now Envigo) born in the CHOP colony maintained at 21°C with a 12:12 light cycle. Food and water were available *ad libitum*. Cages were checked twice daily at 10 a.m. and 6 p.m. and the day of birth was defined as PN0. Pups were weaned at PN21, after behavior testing, and housed 2–3/cage for the remainder of the experiment. The Institutional Animal Care and Use Committees at CHOP approved all animal care and experimental procedures, which follow the guidelines from the National Institutes of Health.

### Experimental Design

Experimental groups were always within a single litter and included: central nerve root compression; peripheral nerve compression, sham operated and naïve controls. Testing experimental groups within litters greatly reduces variability and increases power. No more than one condition was repeated in a single litter for any dependent measure. Surgery was performed on 10-, 14-, 21-, or 28-day old animals. We tested for thermal hyperalgesia and tactile allodynia 1, 7, and 14 days postsurgery (PS1, PS7, and PS14) in the same animals, except for the PN10 animals who showed no or minimum pain at either 1 or 7 days and were not tested further. At each postoperative time point other animals were sacrificed for either immunohistochemistry or quantitative RT-PCR. We were not powered for rigorously testing sex differences, but preliminary analyses showed no differences for either thermal and mechanical behaviors. For PCR, the smaller *N* and uneven distribution of males and females made those comparisons impossible. Therefore, for all subsequent analyses male and female data were combined.

Our primary interest was root compression for comparison to the adult literature [Figure 1A; (50–52)]. We also tested nerve compression because with few exceptions the infant methods have damaged a nerve. Data for the sham root and sham nerve surgeries were very similar. Therefore, to reduce animal numbers, in some litters, a single root or nerve sham surgery was performed rather than both, and the data combined. A separate untreated group served as the non-surgical naïve controls, and withdrawal latencies for the left and right paws were averaged for those controls.

### Surgery

Compression was conducted in fully anesthetized rats aged 10, 14, 21, or 28 days of age. These ages include the human equivalents of newborns through early adolescence (Table 2). All surgical procedures were performed under inhalation anesthesia (4% induction with isoflurane and 2% isoflurane maintenance with oxygen) using aseptic techniques. Breathing rate and hindpaw pinch response were monitored throughout surgery to ensure adequate depth of anesthesia. Rodents were placed in a prone position and any hair on the back of the neck was removed, and the area disinfected using Betadine, followed by alcohol and then covered with a sterile drape. Following a skin incision using a sterile scalpel, the right paraspinal muscles were separated from the spinous processes at the C4–T2 levels. The laminae, facet joint, and spinous process on the right side at C6–C7 were carefully exposed under a surgical microscope. The right transverse process was removed at the C7 level to expose the C7 nerve roots of interest. Central or peripheral nerve compression was *via* surgical vessel microclips and was unilaterally applied to the C7 right mixed nerve or nerve root (Figure 1A). Compression was applied for 15 min and then removed. Microclips have been commonly used in other animal models of radiculopathy (49, 50, 53). Sham procedures were performed in separate groups of rats and involved all surgical procedures except that the nerve or root was not disturbed. After surgery, all wounds were washed with preservative-free sterile saline and closed in two layers (paravertebral muscle layer and skin) with monofilament suture and surgical glue. For the 10- and 14-day old pups, a commercially available nail biter/thumb sucking deterrent was placed near the surgical region to discourage the mother from disturbing the wound. Rats recovered in a cage warmed by a heating pad in room air and closely monitored by the surgeon for up to 1 h following surgery. Typically, most rats recovered from the effects of surgery within 10–20 min. This study of pain precluded the postlesion use of analgesics in all groups. The rats were monitored daily following surgery and did not exhibit any signs of distress or infection.

**Table 2:** **Rough age equivalents between rats and humans**.

Rat	Human
PN10	Newborn
PN14	Early childhood
PN21	Early preadolescent child
PN28	Early adolescent, presexual maturity

*PN is postnatal day*.

### Tests for Thermal Hyperalgesia

Thermal withdrawal latencies were assayed under non-restrained conditions for both forepaws. Each animal was placed singly within an inverted Plexiglas cage upon an elevated glass pane maintained at 30°C (e.g., Hargreaves apparatus). A radiant heat source (24 V halogen lamp focused through a convex lens to a 2 × 4 mm area) beneath the glass was trained on the lateral plantar surface of the forepaw. The heat stimulus and an automatic timer were activated simultaneously. When the rat lifted its paw, the switch simultaneously turned off the stimulus and timer. Cut-off time was set at 20 s to avoid tissue injury.

### Tests for Mechanical Allodynia

The tactile sensitivity for both forepaws was measured as the latency to withdraw the paw to a mechanical probe. Each rat was previously acclimated to the environment and experimenter and gently restrained. In each session, a series of tactile stimuli were placed on the dorsal surface of each forepaw, just behind the interdigital web between the second and third toes. We used differing weighted probes that floated freely [3.3, 5.6, 7.5, 10.1 g; (54, 55)]. Latency to move the paw was measured. Because there were few interactions of the treatment with intensity, we averaged the response latency over the intensities. For all testing procedures, animals were free to remove themselves from the stimulus.

### Dissection

Following testing, subjects were deeply anesthetized with a sodium pentobarbital and when fully unresponsive to pinches and air puffs, they were either transcardially perfused (4% paraformaldehyde) for IHC or decapitated for qPCR. Following perfusion, the spinal cord was dissected out and place overnight in paraformaldehyde. The cervical cord, just rostral to the compression site, was blocked and placed in sucrose prior to cryostat sectioning. For qPCR, the spinal cord was rapidly removed and placed on ice. The cervical enlargement was isolated, and the dorsal cord above the central canal (separate for the ipsilateral and contralateral sides to the compression side) was removed, and frozen at −80°C until assayed.

### Quantitative RT-PCR

We assayed c-fos and a number of markers of immune related function, chemokines, and cytokines by quantitative RT-PCR (Taqman) in the dorsal spinal cord 1, 7, and 14 days postsurgery (except at PN10) in parallel with the behavioral studies. Each was chosen as a marker of immune related processes such as cyclooxygenase 2 (COX-2), toll-like receptor 4 (TLR-4), ionized calcium-binding adapter molecule 1 (IBA-1; for microglia) or Glial fibrillary acidic protein (GFAP; for astrocytes), or because they were inflammatory or anti-inflammatory cytokines and chemokines that have been implicated in chronic or neuropathic pain (see Table 3). Standard methods were used. Briefly, after homogenizing with guanidine-thiocyanate-containing lysis buffer, total RNA was isolated using RNeasy Mini Kit (Qiagen). Traces of DNA are then removed by DNase treatment on the column and the total RNA was eluted in RNase-free water. Five microgram DNase-treated total RNA from each sample was incubated with 50 pmol T7 (dT)24 primers (Affymetrix) at 70°C for 10 min. Using this RNA as a template, single-strand cDNA was synthesized by incubating with Reverse Transcriptase (SuperScript II RT, Invitrogen) as well as first Strand Buffer and 10mM dNTP (Invitrogen) at 42°C for 1 h followed by 70°C for 15 min to denature the enzymes. Newly synthesized ss-cDNA was diluted in pure H_2_O. Real-Time PCR was performed using a StepOne plus machine with TaqMan fast advanced master mix and Taqman primers (all Applied Biosystem), using the “Fast” protocol. The exceptions were IL-4 and IL-10 which were assayed by SYBR Green methods as previously described (56). To quantitate the mRNA changes, we used the ΔΔCt method with GADPH as the reference primer. We have compared GADPH to S18 and β-actin and found identical results previously.

**Table 3:** **Function of immune markers from Figures 4 and 5**.

IBA-1	Marker for macrophage/microglia activation

GFAP	Marker for astrocytes

COX-2	Inducible enzyme for production of prostaglandins

TLR-4	Receptor for gram-negative bacteria (LPS); activates innate immune system

IL-1α	Proinflammatory cytokine

IL-1β	Proinflammatory cytokine

IL-4	Anti-inflammatory cytokine

IL-6	Proinflammatory cytokine

IL-10	Anti-inflammatory cytokine

CCL2 (MCP-1)	Proinflammatory chemokine

CCL3 (MIP-1a)	Proinflammatory chemokine

CCL5 (Rantes)	Proinflammatory chemokine

TNFα	Proinflammatory cytokine

### IHC

We stained 30 μm frozen floating sections for myelin basic protein 1 (Covalence) at a dilution of 1:1,000 using standard ABC protocols (57) as described previously (58). Controls include staining without the primary antibody. We did not quantitate the resulting micrographs.

### Statistical Analysis

For the behaviors, withdrawal latencies were analyzed by two-way ANOVAs to determine overall significant differences. The two factors were lesion type (ipsilateral to compression, contralateral to compression, ipsilateral sham, and naïve animals) and days postsurgery. Because all surgeries were within litters and each animal was tested at each time point, both factors were considered matched values and analyzed by a repeated-measures ANOVA. Tukey’s multiple comparison tests were used to determine individual comparisons of the ipsilateral compression subjects to the naïve animals and to the ipsilateral sham controls. We did not compare the ipsilateral to the contralateral side because in some cases the contralateral side was altered as well, although not significantly. Those data are shown but not used in the statistical comparisons.

### qPCR

The number of cycles for the naïve group (compared to GADPH) was subtracted from each experimental condition in the same litter and analyzed by a two-way ANOVA. Because all treatments were in a single litter, that analysis was repeated measures. Each time point was analyzed separately and Tukey tests for multiple comparisons were between the injured ipsilateral side and the ipsilateral sham control. The data are presented as fold change relative to the naïve animals.

## Results

### Histology

Neither root nor nerve compression induced gross qualitative changes in the dorsal horn structure (Figure 1B). We did not quantitate these results and thus cannot make conclusions about relative changes in myelin or cell density at different ages and times postinjury.

### Behavioral Tests

**Figure 2:**
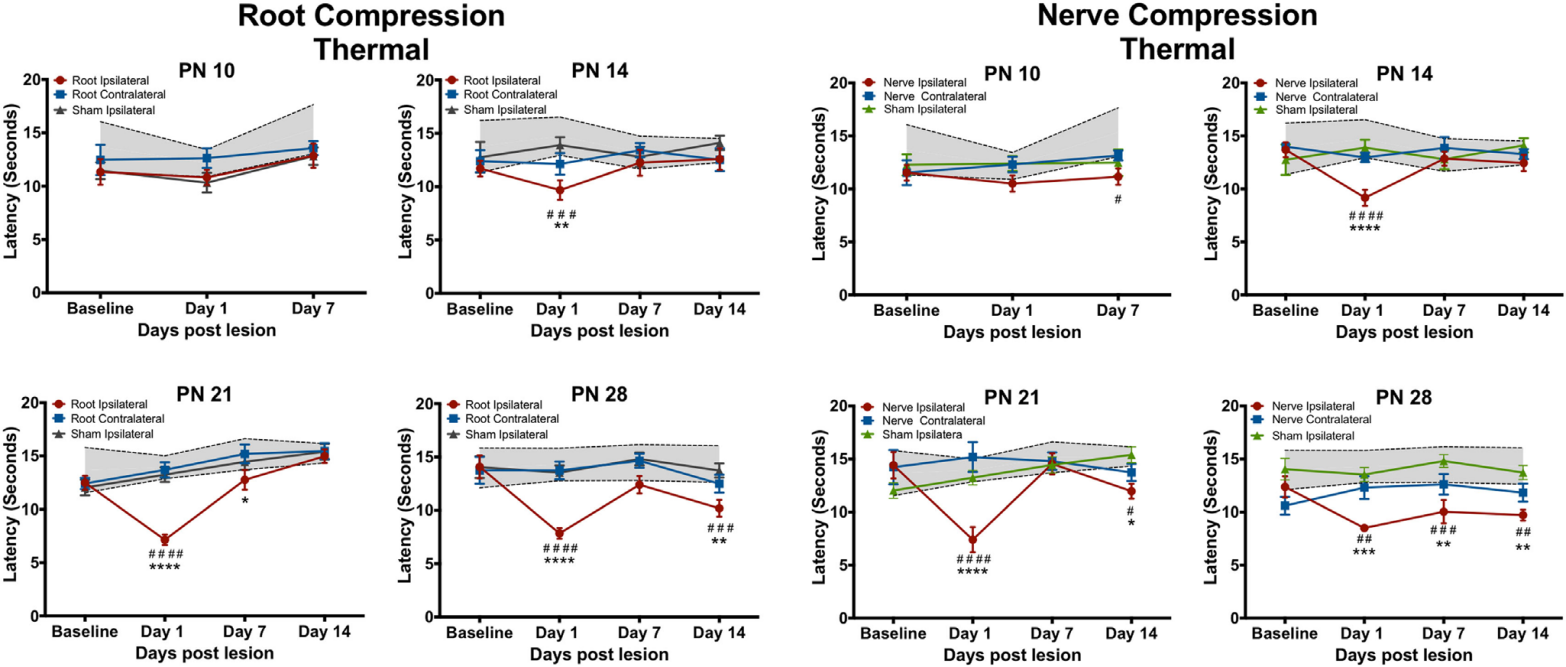
**The change in the forepaw response to the thermal stimulus following root or nerve compression**. The age of surgery is above each panel and the *X*-axis denotes the time of testing postsurgery. The 95% confidence intervals for the naïve controls are denoted by the shaded area between the dashed lines. Significant differences from the naïve subjects are denoted by * and from the forepaw ipsilateral to the sham surgery by #. *N* = 8 except PN14, *N* = 6. *^/#^*p* < 0.05; **^/##^*p* < 0.01; ***^/###^*p* < 0.001; ****^/####^*p* < 0.0001.

**Figure 3:**
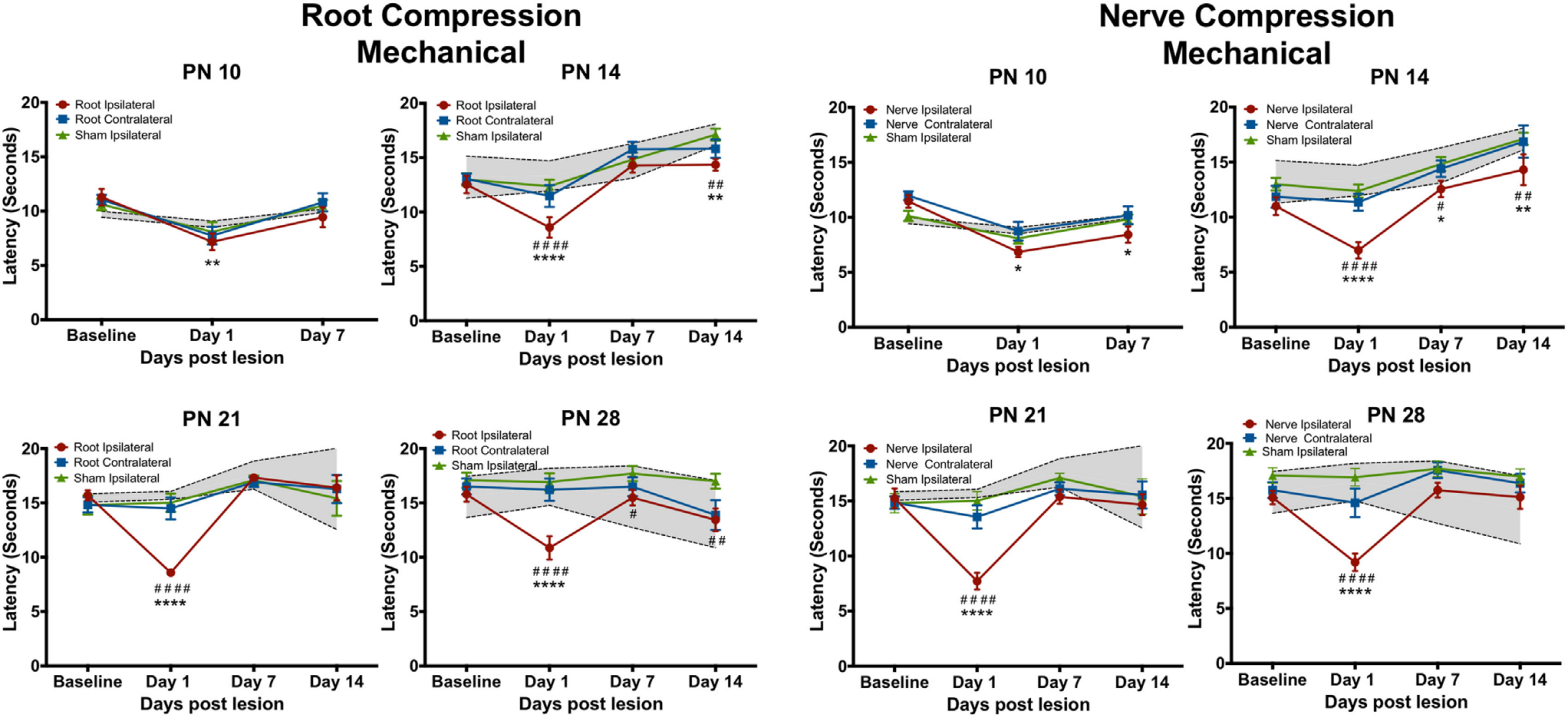
**The change in the forepaw response to the mechanical stimulus following root or nerve compression**. Details are as in Figure 2. *N* = 8 for PN10 and PN28; *N* = 6 for PN14 and PN21.

Figures 2 and 3 show the results of root or nerve compression on the thermal response latency. Comparisons were of the ipsilateral paw of the compressed root or compressed nerve to the ipsilateral paw sham and the naïve controls. In separate analyses, the contralateral side did not differ from either the sham contralateral side or the naïve controls for the thermal test and only at one comparison for the mechanical test (PN14, PS1). *F*-values presented below are for the treatment × postsurgical day interactions. Multiple comparison *p*-values are in Figures 2 and 3.

### Thermal Tests

Neither root nor nerve compression at PN10 changed pain responses compared to the sham control [Root: *F*(6,42) = 0.731, *p* = 0.627; Nerve: *F*(6,42) = 0.998, *p* = 0.439] However, at PN14, PN21, and PN28, both nerve and root injuries produced a large reduction in the thermal response latency 1 day postsurgery [Root/PN14: *F*(9,45) = 1.489, *p* = 0.181; PN21: *F*(9,63) = 6.84, *p* < 0.0001; PN28: *F*(9,63) = 3.691, *p* = 0.001; Nerve/PN14: *F*(9,45) = 3.237, *p* = 0.004; PN21: *F*(9,36) = 5.765, *p* < 0.001; PN28: *F*(9,45) = 1.982, *p* = 0.064]. At PN14, withdrawal latencies following root or nerve injury returned to control levels 7 and 14 days postsurgery. At PN21, the root injuries reduced pain thresholds 1 day postoperatively, returning to control levels at PS7 and PS14. Nerve compression at PN21 showed a slightly different result with a return to control levels at PS7 but a reappearing slight hyperalgesia at PS14. When either nerve or root compression was performed on PN28, the hyperalgesia lasted for 14 days after surgery, the longest time tested.

### Mechanical Tests

The mechanical allodynia results differed slightly from those of the thermal test. In separate analyses, the contralateral side never differed from either the sham contralateral side or the naïve controls, except at PN14, PS1. Again at PN10, there were no effects when compared to the ipsilateral sham control but there was a slight reduction (< 2 s) in latency compared to naïve controls for both root and nerve injury [Root: *F*(6,42) = 3.327, *p* = 0.010; Nerve: *F*(6,42) = 4.491, *p* = 0.001]. At PN14, 21, and 28, there was a short-term increase in sensitivity at PS1 [Root/PN14: *F*(9,45) = 5.315, *p* = 0.002; PN21: *F*(9,36) = 9.915, *p* < 0.0001; PN28: *F*(9,63) = 5.425, *p* < 0.001, *p* = 0.001; Nerve/PN14: *F*(9,45) = 2.756, *p* = 0.012; PN21: *F*(9,36) = 6.056, *p* < 0.001; PN28: *F*(9,63) = 5.425, *p* < 0.001]. Following root injury, the withdrawal latencies returned to baseline levels by PS7 and PS14, regardless of the age of injury, except when performed at PN14. There was a reduction in the latency at PS14, largely because of increased latency in controls at those times that were actually higher than baseline latencies. For the nerve compression, at PN14, there was hyperalgesia at all postsurgical times. When surgery was performed at PN21 or 28, latencies returned to control levels by PS7 and PS14.

### qPCR – Root Compression

**Figure 4:**
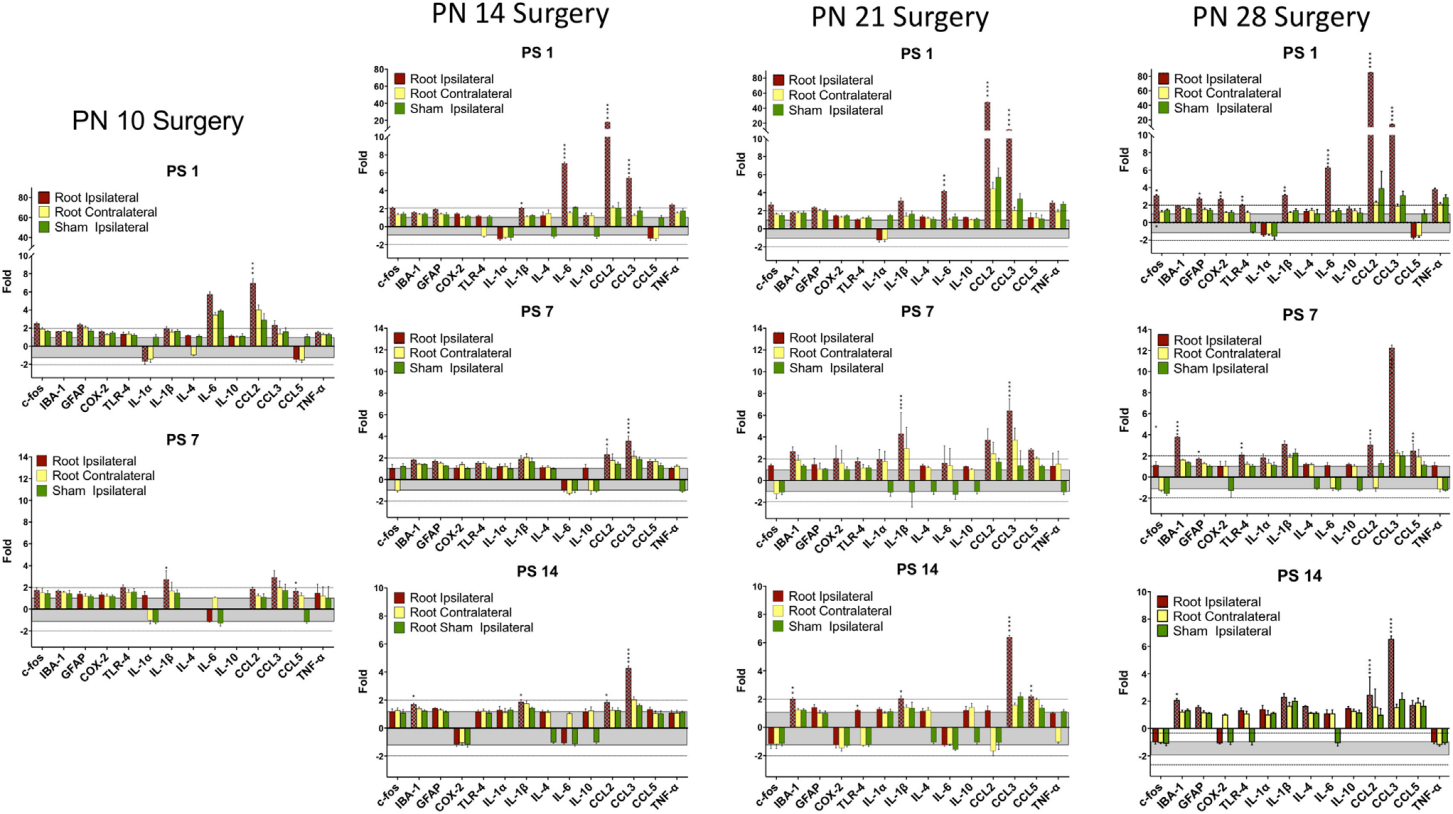
**Change in mRNA for immune related markers, cytokines, and chemokines at different times post-root compression at different ages in the dorsal horn of the spinal cord**. Note the change in the *Y*-axis scale for different postsurgical times. Asterisks denote statistical differences between the side ipsilateral to the compression and the ipsilateral side of the sham animals (see Figure 2). Also shown, but not tested statistically are the dorsal horn data contralateral to the injury. All fold change in the side ipsilateral to the injury are shown by hatched bars. The gray area bounds ±1.0 for which there are no values. The dashed lines are twofold levels. *N* = 4 per group.

**Figure 5:**
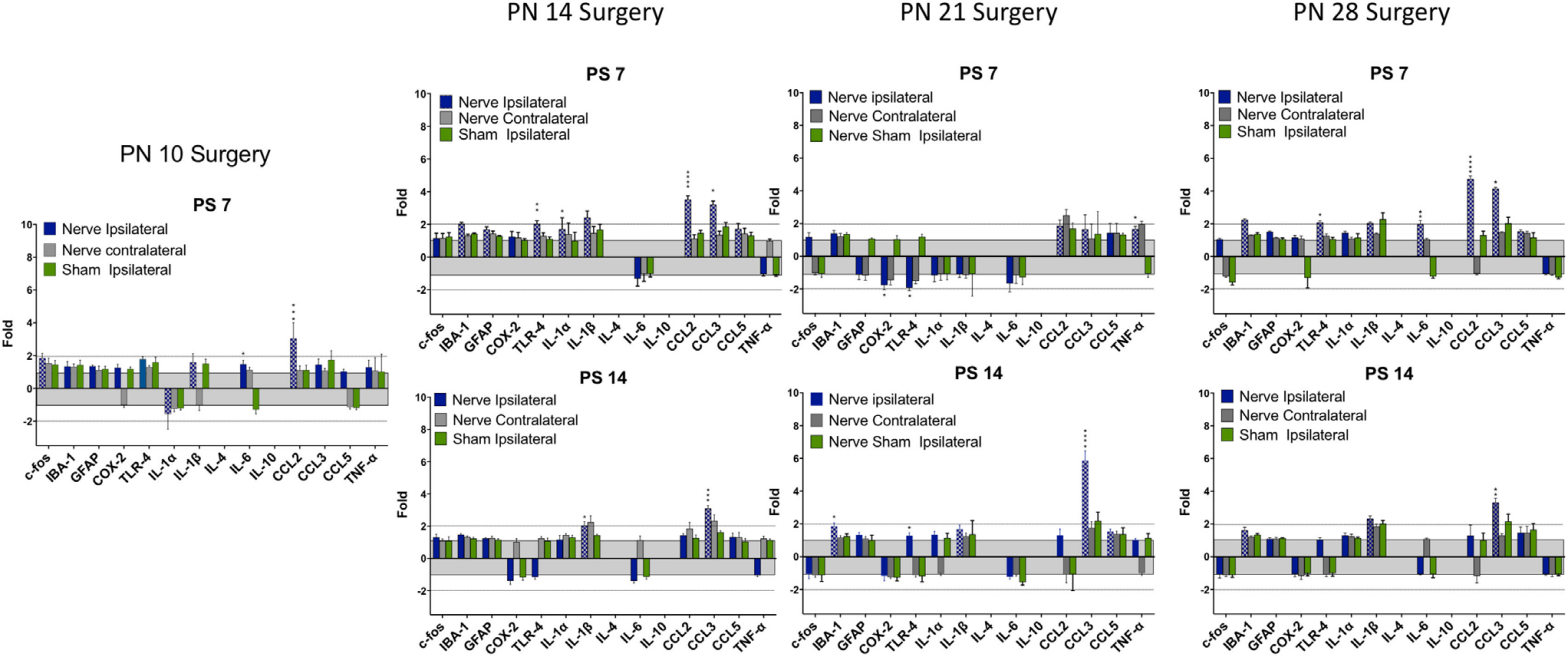
**Change in mRNA for immune related markers, cytokines, and chemokines at different times post-nerve compression at different ages in the dorsal horn of the spinal cord**. *N* = 4 per group. Details as in Figure 4.

There were several patterns that emerged from these data (Table 4; Figures 4 and 5). First, the number and levels of elevated markers were highest at 1 day postsurgery. However, at PN14 and to a lesser degree at PN21, the number and magnitude declined at later time points. The magnitude of changes at PN28 declined over time but the number of significant changes remained constant. Thus, overall activation of cytokines, chemokines, and other immune markers was more prolonged the older the animal at surgery (detailed below).

**Table 4:** **Summary of qPCR results**.

	PN10			PN14					PN21					PN28				
	PS1	PS7		PS1	PS7		PS14		PS1	PS7		PS14		PS1	PS7		PS14	
	R	R	N	R	R	N	R	N	R	R	N	R	N	R	R	N	R	N
c-fos	*													**	*			
IBA-1							*					**	^^^		****		*	
GFAP														*	*			
COX-2											^^^			**				
TLR-4						^^^^					^^^^^	*	^^^	**	**	^^^		
IL-1α						^^^												
IL-1β		*		*			*	^^^		***		*		**				
IL-4																		
IL-6			^^^	****					***					****		^^^^		
IL-10																		
CCL2	***		^^^^^	****	**	^^^^^^	*		****					****	***	^^^^^^	****	^^^^
CCL3				****	****	^^^	****	^^^^^	****	***		****	^^^^^	****	****	^^^	****	
CCL5		*										**			***			
TNFα											^^^							

*R is root compression; N is nerve compression*.

*^/^^*p* < 0.05; **^/^^^*p* < 0.01; ***^/^^^^*p* < 0.001; ****^/^^^^^*p* < 0.0001.

*Note that there are no data for nerve compression at PS1*.

At PN10, only CCL2 was significantly elevated by root compression compared to controls at PS1, and TNFα and CCL5 were significantly elevated at PS7.

By PN14, a number of proinflammatory cytokines were stimulated (IL-1β, IL-6, CCL2, and CCL5). This begins a pattern of consistent activation for CCL2 and CCL3, which were elevated at all postsurgical ages at PN14 and older. This activation declined such that by 7 days postsurgery, there were few differences and only CCL2 and CCL3 were at the twofold change level and significantly different from controls. At PS14, the microglia marker, IBA-1 and IL-1β were significantly elevated in addition to CCL2 and CCL3.

When injury occurred at PN21, 1 day postsurgery, there were significant changes in the IL-6, CCL2, and CCL3 at higher expression levels than at younger ages. The inflammatory chemokines CCL2 and CCL3 were expressed at 10–25-fold compared to controls. Some proinflammatory cytokines/chemokines were also elevated on the contralateral side (IL-1β, CCl2, CCL3, and TNFα) but not to the extent of the injured side. Seven days postsurgery, IL-1β and CCL3 were significantly different from controls and many other immune markers were increased twofold, although not statistically different from controls, where were also elevated. At 14 days postsurgery, there was still elevation of IBA-1, IL-1β, and CCL3.

At PN28, 1 day postsurgery, mRNA for COX-2, FOS, GFAP, TLR4, and the proinflammatory cytokines/chemokines, including IL-1α, IL-1β, IL-6, CCL2, and CCL3 were all increased. At postsurgery day 7, many remained elevated and there was now increased expression of IBA-1 and GFAP. At postsurgery day 14, CCL2, CCL3, and IBA-1 remained significantly elevated. These data were consistent with those of others and confirm a late onset for immune activation following nerve injury.

There were some consistencies among those changes over time. IBA-1, the microglia marker, showed a delayed elevation at PS14 in all three of the older animals. CCL2 and IL-6, in contrast, were elevated at PS1 at the three older ages. Likewise, IL-1β showed greater increases with age. CCL3 was elevated at all time points at PN14 and older. Only when surgery was at PN28, were c-fos, COX-2, GFAP, and TLR-4 were elevated.

### qPCR – Nerve Compression

There were fewer changes at PS7 and PS14 following nerve injury than root injury and the levels were of lower magnitude, with the caveat that we did not assess changes at 1 day postsurgery. Only CCL3 was consistently elevated at PS14 at any age.

## Discussion

Using a single brief compression injury to either the dorsal nerve root or to the nerve, we found an immediate (1 day) thermal hyperalgesia and mechanical allodynia when injury was at 14–28 days of age but minimally at PN10. This response, 1 day after surgery, has often been reported for adult animals in multiple models [e.g., Ref. (59)]. However, consistent and prolonged thermal hyperalgesia was only seen when the surgery was at PN28. Mechanical allodynia was less prolonged lasting beyond 1 day postsurgery only at PN14. It is unclear why this occurred only at PN14, although the behavioral response to the mechanical stimulus was more variable in the older animals.

There were fewer and shorter-lasting changes in dorsal horn expression of proinflammatory cytokines/chemokines when root compression surgery was prior to PN28 with only CCL3, and to a lesser extent, CCL2, showing long lasting overexpression. However, at PN14, PN21, and PN28, IL-6 was upregulated on the first day postsurgery and returned to baseline 1 week postsurgery. Likewise, the microglial marker, IBA-1 was overexpressed at the later postsurgical times when surgery was conducted at those three ages. At PN21 and PN28, there was a particularly striking overexpression for CCL3, which showed a 40–75-fold change 1 day after surgery. At PN28, there were substantially greater numbers and levels of expression, including GFAP, TLR-4, COX-2, the interleukin cytokines, and CCL2 and CCL3. Note that IBA-1 and TLR-4 both increased when the animals were tested at 35 days of age regardless of whether the injury was at PN21 (tested at PS14) or PN28 (tested at PS7). The overexpression for most markers lasted at least 7 days, and CCL2 and CCL3 were continually expressed at high levels through the 14 days of postsurgical testing. These results are consistent with the demonstrated increased immune cell reaction and glial response to nerve injury with age [see Ref. (18) for a recent review].

The proinflammatory cytokines and chemokines are established regulators of neuropathic pain and both mRNA and protein are elevated following nerve injury in adults. CCL2, CCL3, CCL5, IL-1β, and IL-6 are upregulated in damaged nerves, DRGs, and spinal cord dorsal horn (60–63) in variety of models and neuropathic pain is decreased by antagonists, neutralizing antibodies, microglia inhibitors, or in CCL2 deficient mice (62, 64–66). However, in the present study, CCL3 expression was elevated at all ages at PS1 in the root compression and at PS7 in the nerve compression injury, which is not consistent with the appearance of thermal or mechanical hyperalgesia. It is possible that the early CCL2 expression is necessary but not sufficient to drive the behavioral changes.

CCL5 recruits leukocytes to a site of injury in the adult (67) and is upregulated by injury. Reduction of CCL5 by neutralizing antibodies reduces hyperalgesia. In the current experiments, CCL5 was only minimally upregulated root compression surgery at PS21 and PS28, and then only 14 or 7 days after surgery, respectively. CCL5 was not altered by nerve compression in any group. The lack of upregulation of CCL5 is consistent with at least one adult study using the partial sciatic nerve that reported upregulation of CCL1 and CCL3 but not CCL5 (68), although other studies using different models do report upregulation.

Nerve injury upregulates IL-6 and reduction of IL-6 reduces neuropathic pain (65, 69, 70). It has been proposed that TNFα upregulates IL-6 expression *via* a NF-κB pathway (71). Our results are not consistent with that since IL-6 was upregulated early in the root compression animals in the absence of change in TNFα.

Thermal hyperalgesia was similar for both nerve and root compressions with the exception of PN21 where latencies following nerve compression returned to baseline 7 days postsurgery but were lower than controls at PS14. Thermal latencies were significantly reduced at PS7 but returned to control levels at PS14. Mechanical allodynia likewise was similar between the root and nerve compression treatments, but the effects differed from those of the thermal hyperalgesia. There was a slight allodynia at PN10 for the nerve compression compared to naïve rats and for root compression, there was a difference at PS1 following root compression, but again only compared to the naïve animals. When nerve compression was performed at PN14, there was a small but persistent allodynia that lasted until PS14, the last day tested.

There were several differences between the results with the compression model and those with various nerve ligation models (see Table 1). First, thermal hyperalgesia was more prolonged in the PN28-day old surgical animals compared to the mechanical allodynia. In contrast, in adult compression studies, mechanical allodynia is persistent (50). In developmental studies, the delayed appearance of neuropathic pain is specific for a mechanical stimulus, not thermal stimuli (17, 21, 72). There are at least two possible reasons for this. The first is the nature and duration of the injury. The compression as used here is brief whereas in the ligation models the injury is chronic. Thus ligation is a more continuous injury compared to single acute compression. This may be particularly important in infant and young animals where sensory neurons from the DRG are still growing during the first week of life (73–75) perhaps changing the nature of the ligation as the axons grown. Second, the root injury was just proximal to the DRG and the nerve injury just distal to the DRG. As found here, the changes in pain sensitivity were similar between the two injuries but the nerve injury induced fewer changes in immune markers sensitivity particularly at PN28. We know of no root ligation models during early development, but dorsal rhizotomy (76) or dorsal root constrictions (77) produce neuropathic pain in the adult. Although there are no developmental dorsal root constriction models, dorsal rhizotomy in infants does not induce self-mutilation whereas it does in 40% of juveniles and 80% of adults (78). Third, the prior infant injury models induced nerve injury in the hindpaw/lumbar spinal cord whereas the injury described here was forepaw/cervical spinal cord. We know of no data suggesting differences however. Although we did not quantitate anatomical changes in the spinal cord here, future work should directly compare brief compression injury to comparable ligation models, including changes in dorsal horn structure and function to determine if those changes could account for differences among injury models.

We found no injury-induced changes in anti-inflammatory cytokines such as IL-4 and IL-10, despite the elegant data showing that these cytokines are developmentally regulated and actively suppress expression of proinflammatory cytokines/chemokines and neuropathic pain prior to 33 days of age in a spared nerve injury (SNI) model (48). We also found that these cytokines were overexpressed in the untreated infants relative to 42-day old rats. Both IL-4 and IL-10 were about three- to fourfold enriched at PN10–21 compared to adolescents. We did not measure protein levels of release of these cytokines. Thus, it is possible that expression of IL-4 and IL-10, although showing the normal reduction with age, did not reflect the changes following compression that were found in a ligation model (48), although others have reported greater expression of IL-4 in adults than infants using the SNI method (17). Alternatively, the single compression injury, unlike the SNI, may not stimulate the dominant anti-inflammatory response induced in that model.

Finally, our data are consistent with the existent literature that early injury does not induced neuropathic pain, possibly because of less immune activation, and extend those data to a nerve and nerve root compression injury. In the current studies, we did not find evidence of a delayed mechanical allodynia as reported previously (21, 48, 72). Further work will be needed to understand these differences, identifying mechanisms that account for both the similar and different results and to extend them to other assays (e.g., cold allodynia) that also show onset of neuropathic pain after the third week of life in the rodent. Moreover, whether or not there are sex differences in the development of neuropathic pain at these ages is an important question given that there are sex differences in adults and that they are mediated by different components of the immune system [e.g., Ref. (79–82)].

## Author Contributions

GB and BW conceived and designed the study and wrote the manuscript. SW and CW adapted the surgical methods for infants and performed the surgery. SW tested the subjects.

## Conflict of Interest Statement

The authors declare that the research was conducted in the absence of any commercial or financial relationships that could be construed as a potential conflict of interest.
